# Role of EscU auto-cleavage in promoting type III effector translocation into host cells by enteropathogenic *Escherichia coli*

**DOI:** 10.1186/1471-2180-11-205

**Published:** 2011-09-20

**Authors:** Jenny-Lee Thomassin, Xiang He, Nikhil A Thomas

**Affiliations:** 1 Department of Microbiology and Immunology, Dalhousie University, 5850 College Street, P.O. Box 15000, Halifax, Nova Scotia, B3H 4R2 Canada; 2 Department of Microbiology and Immunology, McGill University, 3775 University Street, Montreal, Quebec, H3A 2B4 Canada

## Abstract

**Background:**

Type III secretion systems (T3SS) of bacterial pathogens coordinate effector protein injection into eukaryotic cells. The YscU/FlhB group of proteins comprises members associated with T3SS which undergo a specific auto-cleavage event at a conserved NPTH amino acid sequence. The crystal structure of the C-terminal portion of EscU from enteropathogenic *Escherichia coli *(EPEC) suggests this auto-cleaving protein provides an interface for substrate interactions involved in type III secretion events.

**Results:**

We demonstrate EscU must be auto-cleaved for bacteria to efficiently deliver type III effectors into infected cells. A non-cleaving EscU(N262A) variant supported very low levels of *in vitro *effector secretion. These effector proteins were not able to support EPEC infection of cultured HeLa cells. In contrast, EscU(P263A) was demonstrated to be partially auto-cleaved and moderately restored effector translocation and functionality during EPEC infection, revealing an intermediate phenotype. EscU auto-cleavage was not required for inner membrane association of the T3SS ATPase EscN or the ring forming protein EscJ. In contrast, in the absence of EscU auto-cleavage, inner membrane association of the multicargo type III secretion chaperone CesT was altered suggesting that EscU auto-cleavage supports docking of chaperone-effector complexes at the inner membrane. In support of this interpretation, evidence of novel effector protein breakdown products in secretion assays were linked to the non-cleaved status of EscU(N262A).

**Conclusions:**

These data provide new insight into the role of EscU auto-cleavage in EPEC. The experimental data suggests that EscU auto-cleavage results in a suitable binding interface at the inner membrane that accommodates protein complexes during type III secretion events. The results also demonstrate that altered EPEC genetic backgrounds that display intermediate levels of effector secretion and translocation can be isolated and studied. These genetic backgrounds should be valuable in deciphering sequential and temporal events involved in EPEC type III secretion.

## Background

Type III secretion systems (T3SS) of bacterial pathogens translocate effector proteins into infected cells resulting in a variety of modulations and disruptive actions to host cellular processes. Examples include preventing phagocytosis [1-4], altering Rho signalling [5,6], subverting intracellular membrane trafficking [7-10] and manipulating innate immune responses [11-16].

T3SS are composed of at least 10 conserved proteins [17] some of which are present in multiple copies. Specific protein components form an export apparatus within the inner membrane. A needle complex is formed using the general secretory pathway (sec system) for some of the 'ring' forming components located in the inner and outer bacterial membrane. Cryo-electron microscopy studies of 'needle complexes' isolated from *Salmonella enterica *serovar Typhimurium report a needle-like appendage with a central conduit of approximately 28 Angstroms [18,19]. For EPEC, 'intact' needle complexes have been difficult to isolate [20] and therefore detailed structural information is lacking. A novel difference for EPEC needle complexes is the presence of a polymeric EspA protein filament on top of a basal needle complex [21]. The complete T3SS, composed of the export apparatus and needle complex, then secretes pore and filament forming proteins (EspA, EspB and EspD translocator proteins [22]) and eventually effector proteins, the latter of which are rapidly injected directly into host cells during infection.

A conserved inner membrane protein found in all T3SS is YscU (FlhB homologues). This group of proteins has been the focus of considerable studies owing to an interesting proteolytic activity. Specifically, FlhB/YscU proteins undergo a post-translational intein-like auto-cleavage event at a conserved NPTH amino acid sequence, the result of which leads to proper secretion system functionality [23,24]. Auto-cleavage occurs between the asparagine and proline residues with the resulting polypeptides remaining tightly associated within the bacterial cell [25]. In Enteropathogenic *E. coli *(EPEC), the auto-cleavage mechanism for its YscU homologue, EscU, was elucidated through protein crystallization studies [26]. The reaction mechanism occurs at a type II β-turn and produces a conformational change in EscU, spatially moving the histidine within the NPTH region 180°. It was proposed that this striking conformational change provides a new interface for protein interactions that are required for efficient secretion [26]. In support of this interpretation, a non-cleaving EscU variant (e.g. N262A) did not support type III protein secretion [26]. A soluble C-terminal EscU(P263A) variant also remained un-cleaved in protein crystals, although it was suggested that the reaction mechanism could still occur at elevated pH or with slow kinetics. The protein structures of other EscU homologues (YscU, Spa40) have shown similar auto-cleavage mechanisms [27-29] indicating a remarkable functional importance for this proteolytic event in secretion events. In all cases, the YscU homologue is an essential component of the respective secretory apparatus, however, there is considerable variability amongst bacteria in the secretory phenotypes that are associated with YscU or FlhB auto-cleavage. In the case of *Y. enterocolitica*, non-cleaving YscU variants were found to support secretion of type III effector proteins but not translocator proteins suggesting that YscU auto-cleavage serves to recognize translocators for type III secretion in this pathogen [30]. In two other *Yersinia *species, *Y. pestis *and *Y. pseudotuberculosis*, non-cleaving YscU forms showed dramatic reduction of effector and translocator protein secretion compared to the respective wild type strains suggesting a modulating role for the YscU auto-cleavage event [24,31]. In the plant pathogen *Xanthomonas campestris*, the C-terminal region of HrcU was demonstrated to interact with the putative substrate switching protein HpaC, leading to effector secretion. With respect to flagellum biogenesis, an uncleaved form of FlhB (a YscU homologue) was demonstrated to selectively export only rod/hook-type protein substrates but not filament type substrates [32]. This observation is in line with a modulatory or substrate switching role for FlhB auto-cleavage. From all these studies, it appears that the context of the YscU homologue and its interactions with other secretory components influence T3SS function. It remains that auto-cleavage function is likely contextual and may have specific secretory consequences in different bacteria.

In this study, we provide experimental evidence that EscU auto-cleavage in EPEC promotes effector protein translocation into host cells during infection. In the absence of EscU auto-cleavage, very low levels of effector proteins were secreted as non-functional and abnormal forms. EscU auto-cleavage also promoted efficient membrane association of the multicargo type III chaperone CesT, which has implications for effector delivery into cells during infection.

## Results

### Uncleaved forms of EscU support low levels of translocator and effector protein secretion into culture supernatants

**Figure 1:**
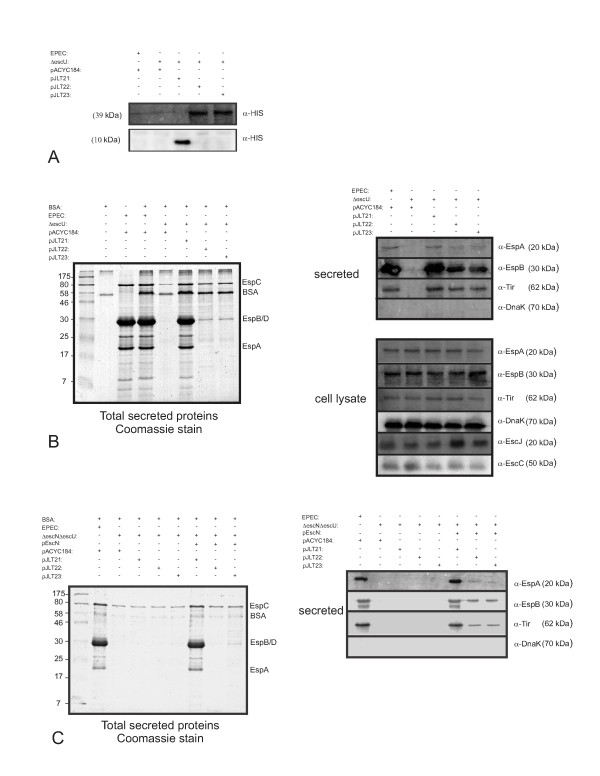
**Efficient translocon and effector secretion is dependent on EscU auto-cleavage**. (A): Immunoblot demonstrating EscU variant cleavage status within whole cell lysates. The blots were imaged separately to get representative signals for the auto-cleavage products. A longer exposure was used for the 39 kDa protein species. (B) Left: trans-complementation of *ΔescU *with pJLT21 restores EspA (~20 kDa), EspB and EspD (co-migrate ~30 kDa) secretion to wild type levels (the identity of these dominant protein species has been previously determined using protein sequencing [36], and here by immunoblotting [right panel]). EspC is an abundant type 5 secreted protein. Bovine serum albumin (BSA) was added to collected secreted protein fractions as a carrier protein to assist in the precipitation of proteins. A molecular weight standard is in the left most lane. Right: immunoblot analyses of secreted protein and whole cell lysate fractions from bacterial strains used in panel A (as indicated). The respective secreted protein fractions were diluted 20 fold prior to SDS-PAGE. (C) Left: secreted protein fractions derived from Δ*escN*Δ*escU *double mutant strains with the indicated plasmids. Right: Immunoblot analysis of secreted protein fractions. DnaK, an abundant non-secreted cytoplasmic protein, was used as a gel loading control (when needed) or to assess cytoplasmic contamination of secreted fractions or non-specific bacterial lysis. All samples were diluted 20 fold as in panel B. All experiments within the panels were performed twice and representative images are shown.

Based on previous protein crystallography studies [26], we generated three recombinant plasmids that encode auto-cleaved or uncleaved histidine tagged forms of EscU (39 kDa) (see Materials and Methods). EscU-HIS (pJLT21), EscU(N262A)-HIS (pJLT22) and EscU(P263A)-HIS (pJLT23) were created for initial characterization studies. Unlike *Shigella *species where Congo Red is used to 'induce' *in vitro *type III secretion [33], culturing EPEC in DMEM 'induces' type III secretion [34,35]. After culturing for 6 hours in DMEM, whole cell lysates and culture supernatants were collected from Δ*escU *strains harbouring pJLT21, pJLT22 and pJLT23. EscU auto-cleavage at the NPTH catalytic site is predicted to produce an 89 amino acid C-terminal product of 10.3 kDa. Immunoblotting whole cell lysates indicated that EscU-HIS was auto-cleaved due to the detection of an approximately 10 kDa species with anti-HIS antibodies (Figure 1A). A longer immunoblot exposure did not reveal any uncleaved EscU (39 kDa) suggesting complete auto-cleavage. In contrast, Δ*escU *whole cell lysates containing EscU(N262A) or EscU(P263A) produced a 39 kDa species detected by anti-HIS antibodies, a molecular weight consistent with uncleaved (intact) EscU.

To further characterize these strains, the respective culture supernatant fractions were evaluated. Under these growth conditions, four predominant protein species are routinely detected in secretion fractions and have been identified using protein micro-sequencing [36]. These include EspA (predicted molecular mass of 20.5 kDa, filamentous translocon protein [37], EspB (predicted molecular mass of 33 kDa, YopD orthologue), EspD (predicted molecular mass of 39.5 kDa, YopB orthologue) and EspC (predicted molecular mass 140 kDa, secreted by the type V secretion pathway). In contrast, low amounts of Tir and other type III effectors are secreted under these conditions but can be detected using immunoblotting approaches. As expected, Δ*escU *expressing EscU-HIS restored EspA, EspB and Tir protein secretion back to wild type EPEC levels (Figure 1B). Δ*escU *expressing either EscU(N262A) or EscU(P263A) had visibly lower amounts of protein species in their respective secretory profiles, however, a notable ~30kDa protein species was detected by Coomassie staining and could represent low levels of either EspB or EspD (predicted molecular masses of 33 and 39.6 kDa respectively). Immunoblotting with anti-EspA, anti-EspB and anti-Tir antibodies demonstrated reduced levels of EspA (~20%), EspB (~20%) and Tir (~70%) from Δ*escU *bacteria expressing either EscU(N262A) or EscU(P263A) relative to EscU (as determined by densitometric analyses). Immunoblotting the whole cell lysates of these strains demonstrated equal steady state amounts of EspA, EspB and Tir were present, ruling out the possibility of intracellular protein expression differences. Immunoblotting the same whole cell lysate samples with anti-EscC and anti-EscJ antibodies revealed equal amounts of the type III secretion apparatus ring forming proteins EscC and EscJ. This latter result indicates that EscC and EscJ protein expression levels are not altered by the cleavage status of EscU.

An *ΔescNΔescU *double mutant was generated to investigate if non-specific leakage from bacterial cells was occurring (perhaps due to overexpression of EscU or multi-copy effects). In the absence of EscN, the ATPase of the EPEC T3SS, type III secretion does not occur [38]. EspA, EspB and Tir were not observed in the secreted sample from the Δ*escN*Δ*escU *double mutant by Coomassie staining (Figure 1C). Immunoblotting using antibodies against EspA, EspB and Tir did not detect these proteins in the Δ*escN*Δ*escU *secretion fraction. Genetic complementation of *ΔescNΔescU *with plasmids expressing wild type EscN and EscU restored the secretion of EspA, EspB and Tir to wild type levels indicating that this double mutant strain could be rescued with multicopy plasmids expressing the appropriate proteins. Complementation of *ΔescNΔescU *with plasmids pJLT21, pJLT22 and pJLT23 (in the absence of pEscN) did not result in EspA, EspB and Tir secretion as assayed by Coomassie staining and immunoblotting (Figure 1C). Based on these data, the small amount of EspA, EspB and Tir in culture supernatants for Δ*escU*/pJLT22 and Δ*escU*/pJLT23 (Figure 1B and 1C) was due to EscU(N262A) or EscU(P263A) expression, and was EscN dependant. Importantly, plasmid mediated genetic complementation does not introduce leakage artefacts to the experimental system.

### The 10 kDa EscU auto-cleavage product is membrane associated

The observation that uncleaved forms of EscU support very low levels of type III translocon and effector protein secretion was unexpected since EscU auto-cleavage has been suggested to provide a binding interface for protein substrate recognition at the base of the T3SS [26]. We therefore set out to evaluate the cleavage state of our EscU variants within sub-cellular fractions enriched for T3SS needle complexes.

**Figure 2:**
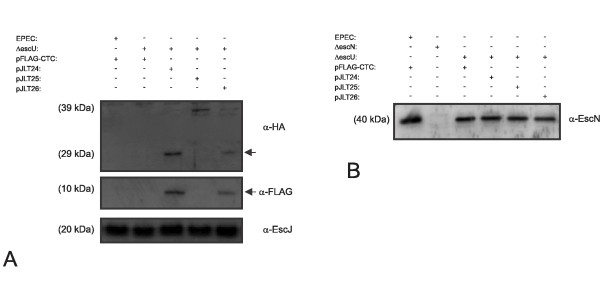
**EscU auto-cleavage results in a 10 kDa C-terminal product that is membrane associated in EPEC**. (A) Isolated membrane fractions were probed with anti-HA and anti-FLAG antibodies to assess EscU auto-cleavage status. Membrane localization of EscJ is unchanged in *escU *null mutants (lane 2) and therefore this protein served as an internal control for the individual membrane fractions. The approximate 10 kDa C-terminal EscU auto-cleavage product (detected with anti-FLAG antibodies) along with the 29 kDa HA-tagged N-terminal product (detected with anti-HA antibodies) both partitioned to the membrane fraction (denoted by arrows). Uncleaved EscU is also membrane associated and appeared as a 39 kDa species. (B) The same membrane fractions were probed with anti-EscN antibodies to detect membrane associated EscN levels. A Δ*escN *mutant membrane preparation was included to demonstrate the specificity of the antibody.

To assess EscU auto-cleavage and to detect post-auto-cleavage products, we generated double tagged recombinant EscU forms. A hemagglutinin (HA) tag was fused to the N terminus and a FLAG tag was fused to the C-terminus of EscU. Using this strategy, wild type EscU auto-cleavage is predicted to produce a 29 kDa transmembrane polypeptide that can be recognized by anti-HA antibodies and a 10 kDa cytoplasmic polypeptide (amino acids 263-345) that can be recognized by anti-FLAG antibodies. Δ*escU*/pJLT24 (expressing HA-EscU-FLAG) demonstrated a wild type EPEC secretion pattern indicating that the presence of HA and FLAG tags did not inhibit EscU function (data not shown). A sub-cellular fractionation procedure to produce a membrane fraction enriched for T3SS needle complexes [39] was then used to evaluate the double tagged protein constructs in the *escU *null mutant. The membrane preparation derived from Δ*escU*/pJLT24 was probed with anti-HA antibodies and anti-FLAG antibodies which detected 29 and 10 kDa polypeptide species respectively (Figure 2). These polypeptide species are in complete agreement with the predicted sizes of EscU auto-cleavage products. No full-length EscU (39 kDa) was detected in the Δ*escU*/pJLT24 membrane fraction, suggesting complete auto-cleavage had occurred under these conditions. EscU(N262A) was detected exclusively at 39 kDa with anti-HA antibodies. Interestingly, EscU(P263A) appeared as a 39 kDa polypeptide along with a 29 kDa and 10 kDa polypeptides detected by anti-HA antibodies and anti-FLAG antibodies respectively. These data demonstrate that the EscU 29 and10 kDa auto-cleavage products localized to membrane fractions enriched for T3SS needle complexes and are in agreement with the crystal structure soluble domain interactions previously reported [26]. In addition, plasmid encoded EscU(P263A) is auto-cleaved in EPEC albeit at reduced levels compared to normal EscU.

The formation of functional T3SS needle complexes is believed to be a multistep process. For EPEC, T3SS needle complexes are less well characterized than those of *Salmonella *and *Shigella *species. Purified EPEC T3SS needle complex preparations often lack certain protein components that are highly conserved in all systems and hence expected to be part of a 'complete' T3SS needle complex. For example EscF, the putative needle protein has not been detected in highly purified EPEC needle preparations [20]. Antibodies to EscJ and EscN [39] were used to probe membrane fractions to assess the expression levels of these proteins. No change in the amount of cell envelope associated EscJ or EscN was observed in ΔescU bacteria expressing any of the EscU variants (Figure 2A and 2B). These data indicate that EscU auto-cleavage is not essential for EscN and EscJ localization to the cell envelope. In certain *Yersinia *and *Shigella *mutant genetic backgrounds and under defined growth conditions, the T3SS needle proteins (YscF or MxiH) are secreted in higher amounts [30,31,40] Since no one has detected polymerized EscF (needle protein) in purified EPEC needle complexes or secreted protein fractions, we are currently unable to assess whether EscU auto-cleavage is required for EscF polymerization or EscF protein secretion (see discussion).

### EscU auto-cleavage is necessary for functional translocation of type III effector proteins into human cells

**Figure 3:**
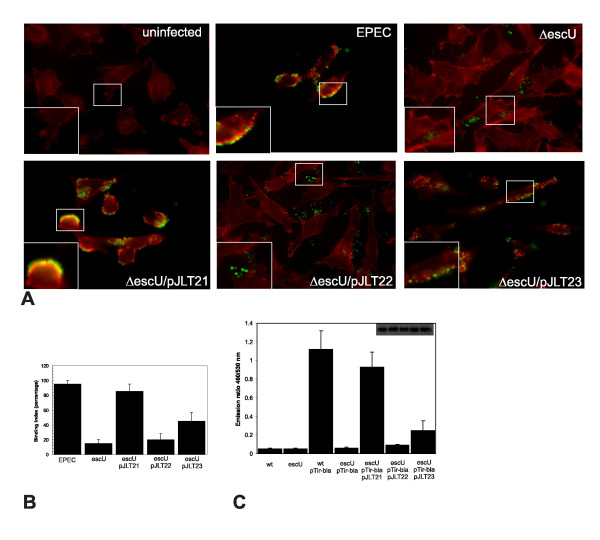
**EscU auto-cleavage is required for Tir translocation, actin pedestal formation and maximal intimate EPEC adherence**. (A) Fluorescent microscopy images of HeLa cells following a three-hour infection with various EPEC strains. Phalloidin staining (red) was used to detect F-actin. All EPEC strains contain a plasmid that encodes GFP (green). Note the strong F-actin enrichment (red signal) within the boxed insets. This experiment was performed twice and representative merged images are shown. (B) Quantification of intimately adherent bacteria using a binding index. The bacterial binding index was defined as the percentage of cells with at least five bound bacteria that co-localized to actin pedestals. At least 50 cells were counted per sample. (C) A Tir-TEM1 effector translocation assay was used to quantify translocation levels during infection of HeLa cells. HeLa cells were infected with the indicated bacterial strains, washed twice to remove non-adherent bacteria and then loaded with the cell permeable fluorescent β-lactamase substrate CCF2/AM. Blue and green (460 and 530 nm) signals were detected with a plate reader and the fluorescence ratio (460/530 nm) corrected for background is shown for the indicated strains. An immunoblot of whole cell lysates with anti-TEM1 antibodies demonstrated equivalent amounts of β-lactamase in the five strains with pTir-bla (inset). The presented translocation assay data are averages of triplicate values of the results from three independent experiments.

The role of EscU auto-cleavage in effector injection during EPEC infection has not been evaluated. We therefore set out to evaluate the role of EscU auto-cleavage during EPEC infection of human cells. We used C-terminal HIS tagged EscU forms for these experiments as we noted the complementation efficiency for these constructs were better than the dual HA and FLAG tagged constructs (data not shown). *escU *mutants expressing EscU-HIS variants were tested for their ability to translocate (inject) the effector Tir into human cells. While the EPEC bundle forming pilus (BFP) is known to mediate early and initial adherence to host cells during infection, T3SS mediated Tir translocation into host cells is required for intimate EPEC adherence (mediated by a Tir/Intimin interaction). In addition, Tir translocation results in F-actin 'pedestal' structures directly beneath adherent bacteria. As expected after a three-hour infection, it was found that the Δ*escU *infection had markedly fewer bacteria intimately associated to HeLa cells (compared to the wild type infection) and could not induce host cell F-actin rearrangement (Figure 3A). Infection with Δ*escU*/pJLT21 fully restored intimate adherence and F-actin pedestal structures to wild type levels, indicating that EscU is required for pedestal formation. The EscU(N262A) variant encoded by Δ*escU*/pJLT22 had similar defects in intimate adherence and pedestal formation as Δ*escU *(Figure 3A). In contrast, EscU(P263A) supported an apparent increase in bacterial intimate adherence and formed short actin pedestals (see inset). Notably all strains express BFP, suggesting that the intimate adherence differences are related to T3SS and EscU function. We further quantified the number of intimately adherent bacteria by microscopic counts. These analyses revealed a significant deficiency in intimate adherence for both the *escU *null mutant and *escU *expressing EscU(N262A) (Figure 3B).

To further support the Tir injection and actin pedestal observations, we employed a Tir-TEM-1 β-lactamase fusion protein (expressed in EPEC and Δ*escU *strains) to report on Tir translocation. This approach uses living cells loaded with a fluorescent substrate that can be cleaved by β-lactamase and has been used in EPEC/EHEC/*Citrobacter *to quantitatively monitor type III effector translocation [41-45]. Using this approach, a Tir-TEM-1 fusion protein was translocated by wild type EPEC but not Δ*escU *(Figure 3C). Δ*escU/*pJLT21 demonstrated translocation of Tir-TEM-1 near wild type levels while Δ*escU*/pJLT23 supported significantly less translocation albeit above Δ*escU *levels. Δ*escU*/pJLT22 was unable to support Tir-TEM1 translocation and appeared similar to Δ*escU*. These results demonstrate that EPEC strains with auto-cleaved forms of EscU supported the translocation of Tir-TEM-1 fusion proteins into infected HeLa cells whereas strains with uncleaved EscU or the absence of EscU did not.

### In the absence of EscU auto-cleavage, novel Tir polypeptides are detected in culture supernatants

**Figure 4:**
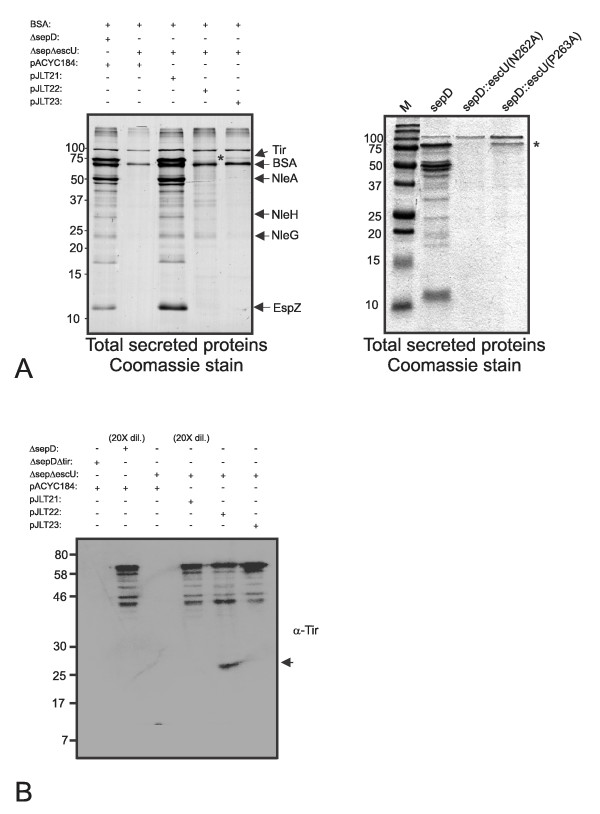
**EscU auto-cleavage is required for efficient and stable effector secretion in an EPEC Δ*sepD *genetic background**. (A) Left: Trans-complementation of *ΔsepDΔescU *with pJLT21 restored secretion of effectors to Δ*sepD *levels while *ΔsepDΔescU/*pJLT22 did not restore normal effector secretion. *ΔsepDΔescU/*pJLT23 secreted a protein with an apparent molecular mass similar to Tir (asterisk). The dominant effector proteins are labelled and have been previously identified using mass spectrometry analyses [35]. Purified BSA was added to collected secreted fractions and served to aid in protein precipitation. Right: genomic integration of mutant *escU *alleles (cis-complementation, single copy) produces the same secretion phenotypes as the plasmid trans-complemented *escU *strains. Total secreted proteins were visualized by Coomassie G-250 staining. (B) Secreted protein preparations were analyzed by immunoblot with anti-Tir antibodies. Due to the abundance of secreted Tir in Δ*sepD *and Δ*sepD*Δ*escU*/pJLT21, (see Coomassie stain in panel A), only these samples were diluted 20 fold for immunoblotting purposes while the others were undiluted. A Δ*sepD*Δ*tir *strain (undiluted) was included to show the specificity of the anti-Tir antibodies. Lower molecular weight protein species are therefore Tir breakdown products that were consistently observed and recognized by the anti-Tir antibodies. A novel Tir polypeptide, indicated by an arrow, was exclusively detected in the lane containing secreted proteins derived from *ΔsepDΔescU/*pJLT22.

The HeLa cell infection experiments established a substantial role for EscU auto-cleavage in Tir and presumably other type III effector injection by EPEC. The *in vitro *secretion assay experiments shown in Figure 1 reveal predominant EPEC translocon protein secretion (EspABD) and very low levels of effector proteins. In contrast, EPEC *sepD *mutants are known to hypersecrete abundant levels of type III effector proteins under the same growth conditions, including Tir, NleA, NleH, NleG and EspZ among others [35,39] (also see Figure 4A). We reasoned that the Δ*sepD *EPEC strain would be a suitable genetic background to gain some insight into the role of EscU auto-cleavage with respect to *in vitro *type III effector secretion. A Δ*sepD*Δ*escU *double mutant was generated and grown under secretion inducing conditions followed by collection of the secreted protein fractions. The secreted protein fraction derived from Δ*sepD*Δ*escU *was visibly lacking many protein species compared to that of Δ*sepD *(Figure 4A). Trans-complementation of Δ*sepD*Δ*escU *with pJLT21 restored secretion back to that of Δ*sepD *with respect to protein amounts and profile. In contrast, the Δ*sepD*Δ*escU*/pJLT22 did not restore a Δ*sepD *secretion profile. Interestingly, Δ*sepD*Δ*escU*/pJLT23 secreted low amounts of a protein species with a similar apparent molecular mass as Tir (Figure 4A). Untagged cis-complemented *sepD*::*escU(N262A) *and *sepD*::*escU(P263A) *strains (expressing the respective *escU *allele from the chromosome) were generated by allelic exchange and were found to produce the same secretion profile as the respective plasmid complemented strains (Figure 4A). Immunoblotting with monoclonal anti-Tir antibodies revealed that Tir secretion occurred at variable levels when EscU or EscU variants were expressed although for EscU(N262A), a novel lower molecular weight polypeptide was detected with anti-Tir antibodies (Figure 4B). This novel polypeptide species was consistently absent from Δ*sepD*Δ*escU*/pJLT21 or pJLT23 and the parent Δ*sepD *strain.

### CesT membrane localization is altered in the absence of EscU auto-cleavage

**Figure 5:**
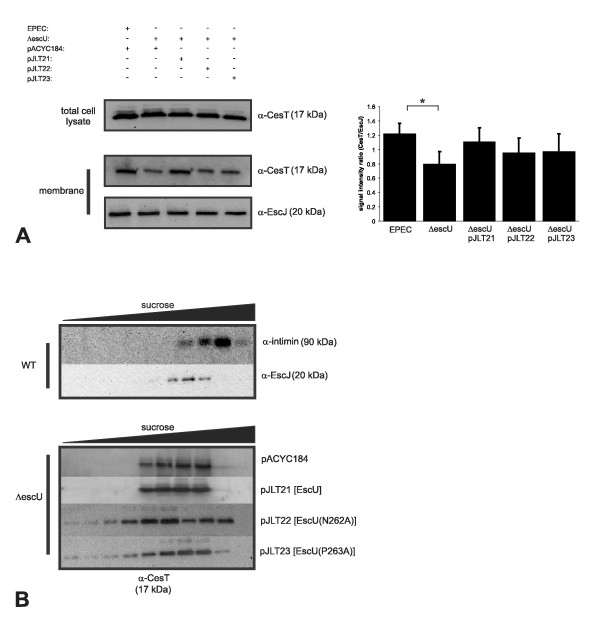
**CesT membrane association is reduced in the absence or with limited EscU auto-cleavage**. (A) Total cell lysates and membrane fractions were probed with anti-CesT antibodies to assess CesT protein levels. The membrane fraction immunoblot was subjected to quantification of band intensity (chemiluminescent signals) to measure CesT protein levels relative to EscJ. EscJ forms a multimeric ring like structure (independent of EscU) and localizes to the inner membrane. For quantification via densitometry, the immunoblots were probed with antibodies and then simultaneously imaged using an exposure time within an empirically determined linear range of signal detection. The densitometry values are averages from three independent experiments and are expressed as a ratio of CesT/EscJ signals as assayed by Quantity One software. A dependent, match paired student's t test was used to assess statistical significance between values (denoted by an asterisk). A representative immunoblot from the experiments is shown. (B) Sucrose density gradient fractionation of membrane preparations from the indicated strains. EscJ and intimin are known inner and outer membrane proteins and their immune-detection served to indicate fractions enriched for inner and outer membranes separated upon ultracentrifugation. Note the altered distribution of CesT in the presence of EscU(N262A) and EscU(P263A).

**Figure 6:**
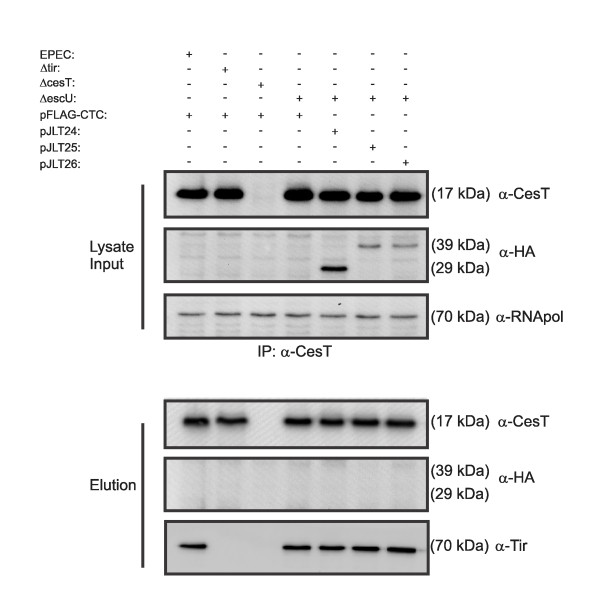
**EscU or EscU variants from EPEC lysates do not co-purify with immunoprecipitated CesT**. Cell lysates were generated from the indicated bacterial strains and exposed to anti-CesT antibodies in a co-immunoprecipitation experiment. The lysate inputs were probed with the indicated antibodies (top panel). Anti-RNA polymerase antibodies were used to detect RNA polymerase amounts within the lysates which are expected to be equivalent. The elution fractions were probed with the indicated antibodies. *tir *and *cesT *null mutants were included as control strains in the experiment. Note that Tir is unstable in the absence of CesT and therefore was not detected in the elution fraction. The lane designations apply to all the panels.

In a previous report, we have demonstrated that the multicargo type III chaperone CesT mediates effector 'docking' at the inner membrane in an EscN-dependent manner [39]. CesT is also required for Tir stability in the EPEC cytoplasm [46,47] and mediates efficient secretion of at least 9 type III effectors [39]. It has also been demonstrated that CesT contributes to effector translocation [42,43]. The detection of a small novel Tir polypeptide in culture supernatants derived from bacteria expressing EscU(N262A) suggested that CesT-Tir interactions may be altered in the absence of auto-cleavage. We therefore set out to investigate CesT-Tir, CesT-EscU interactions in context of EscU auto-cleavage using bacteria that expressed HA-tagged EscU variants. Total cell lysates and membrane preparations were generated from the Δ*escU *mutant expressing either EscU, EscU(N262A) or EscU(P263A) followed by SDS-PAGE and immunoblotting analyses. Total CesT levels were unchanged in all the strains, indicating that EscU auto-cleavage does not influence CesT protein expression or stability (Figure 5). As reported previously [39], CesT was detected within the membrane fraction for wild type EPEC (Figure 5). Band intensity (chemiluminescent signals) was quantified using densitometry normalized to EscJ levels within the same membrane fraction. A reduced amount of membrane associated CesT was observed for Δ*escU *and Δ*escU *expressing either EscU(N262A) or EscU(P263A), as determined by densitometric analyses. The reduced amount was statistically significant for the *escU *null mutant compared to wild type EPEC, although this significance did not extend to the EscU variants. Next, the membrane fractions were subjected to sucrose gradient fractionation to assess CesT membrane localization patterns. EscJ and intimin are inner and outer membrane proteins respectively and hence served to identify inner and outer membrane enriched fractions. For Δ*escU *expressing HA-EscU-FLAG, a strong enrichment of CesT was found within inner membrane fractions. In contrast, HA-EscU(262)-FLAG and HA-EscU(263)-FLAG showed a more diffuse pattern of CesT membrane association, with a considerable amount of CesT protein localizing to less dense fractions within the gradient. These observations suggested that CesT function could be altered or less efficient in the absence of EscU auto-cleavage. We therefore carried out a co-immunoprecipitation assay, using anti-CesT antibodies, to assess CesT-effector interactions. Moreover, it has been shown that HpaB, a type III chaperone, interacts with HrcU [48] (EscU homologue) and hence we asked whether CesT interacts with EscU. Affinity purified anti-CesT antibodies co-immunoprecipitated equal amounts of Tir from all bacterial lysates (Figure 6). This was expected, since CesT is required for Tir stability [46,47], and an earlier result that showed equal steady state levels of Tir in whole cell lysates expressing EscU variants (Figure 1). In contrast, both auto-cleaved and un-cleaved forms of EscU were not co-immunoprecipitated with anti-CesT antibodies.

Taken together, these data indicate that total CesT membrane levels were not statistically different for EscU variant expressing strains, although the nature of CesT association with the inner membrane was altered in the absence or with limited EscU auto-cleavage. CesT retained normal effector binding function in the absence of EscU auto-cleavage and EscU did not co-immunoprecipitate with CesT.

## Discussion

The T3SS is one of the most complex secretory systems in prokaryotic biology, being composed of at least 10 conserved protein components [17]. The YscU/FlhB proteins have been studied in considerable detail, although the phenotypes associated with secretion are highly variable among bacteria and even within the same species [24,30-32,49,50]. The intein-like auto-cleavage mechanism of EscU was previously elucidated through protein crystallography studies. It was proposed that EscU auto-cleavage likely results in an interface for important protein interactions for type III secretion. In this study, we provide evidence to suggest that EscU auto-cleavage supports efficient type III effector translocation. We also observed that the multicargo type III chaperone CesT was less efficiently associated with the inner membrane (Figure 6), which may partly explain the deficiency in type III effector translocation. The data extend previous findings pertaining to EscU auto-cleavage and suggest a critical role for EscU auto-cleavage in preparing the secretory apparatus to accept chaperone-effector complexes.

Previous studies have demonstrated that *escU *null mutants are completely deficient in secreting proteins via the T3SS pathway [26,51]. This phenotype holds true in other pathogens with T3SS and therefore highlights the absolute requirement of YscU/FlhB proteins in type III secretion events. Very little is known regarding how substrate proteins engage the EPEC T3SS export apparatus (EscRSTUV) located in the inner membrane. A non-cleaving EscU(N262A) variant still supported the secretion of Tir although at least one novel Tir derived polypeptide (possibly due to degradation) was linked to the uncleaved state of EscU(N262A). In contrast, bacteria expressing EscU(P263A) did not display novel Tir degradation products which suggests that its low level auto-cleavage is sufficient to support Tir stability and secretion. We did observe reduced levels of membrane associated CesT in the absence of EscU auto-cleavage, although the result was not statistically significant. Our efforts to further explore this observation using other approaches included separating membrane preparations by sucrose density gradients. These analyses revealed that CesT membrane association is less stable without or with minimal EscU auto-cleavage (Figure 5B). Sucrose gradient membrane fractionation is a challenging biochemical technique, and gradient to gradient comparisons and exact gradient reproducibility are difficult. We are in the process of developing *in situ *approaches such as fluorescence energy transfer (FRET) to better assess protein interaction at the base of the T3SS. Nonetheless, the presented experiments provide a framework for future experiments and demonstrate that the presence of auto-cleaved EscU supported localized CesT membrane association.

Recently, Galan and co-workers reported that type III chaperones associate with a 'sorting platform' within the cytoplasm and that this chaperone association influences secretory events [52]. A platform has yet to be identified in EPEC, although it is possible that within our experimental system, CesT may be mis-localized leading to aberrant type III effector secretion. The observation of degraded Tir due to EscU(N262A) (Figure 5B) is in line with this interpretation. Furthermore, the short actin pedestals directly underneath adherent EscU(P263A) expressing bacteria which showed reduced auto-cleavage levels is consistent with this view. Since EPEC Tir injection strictly requires a EspABD translocon [53-56] and EspA filament formation requires EscF expression [57], EscU(P263A) likely supports the formation of a functional T3SS needle apparatus (at reduced levels) formed by the putative needle protein EscF and the translocon proteins EspA, EspB and EspD. We performed three different, yet complimentary infection assays (immunofluorescent microscopy, sub-cellular fractionation and effector β-lactamase fusions) to characterize EscU(P263A) and consistently identified intermediate effector translocation levels. Interestingly, a similar intermediate phenotype was observed for a *Salmonella flhB *null mutant expressing a slow cleaving FlhB(P270A) protein where cells were weakly motile and exported reduced amounts of flagellin [32].

Chaperone-effector complex docking at the inner membrane has been reported for many T3SS [58,59]. We have previously demonstrated that CesT inner membrane association is aided by the presence of the T3SS ATPase EscN [39]. The data cannot rule out the possibility that the EPEC T3SS export apparatus may be structurally impaired or malformed in the presence of uncleaved EscU although it has been demonstrated that un-cleaved forms of EscU can fold correctly [26]. The levels of EscN (T3SS ATPase) were unchanged in Δ*escU *bacteria expressing uncleaved or partially uncleaved forms of EscU (Figure 2B). Since bacteria expressing EscU(P263A) did support effector translocation, albeit at a reduced level, a functional T3SS export apparatus was likely assembled even though EscU(P263A) was only partially auto-cleaved. In support of this, within *S. typhimurium*, uncleaved SpaS (EscU homologue) still supported the formation of a high order export apparatus - needle complex composed of at least 10 proteins as shown by blue native (BN) PAGE of enriched needle complex containing fractions [60].

A number of studies have reported on specific protein-protein interactions important for T3SS function. Auto-cleavage of HrcU (an EscU homologue in *Xanthomonas*) promoted an interaction between the ATPase HrcN, and the C-terminal cleavage product of HrcU [48]. The global T3S chaperone HpaB was also shown to interact with HrcN and the full-length form of HrcU. Co-immunoprecipitation experiments using EPEC lysates and anti-CesT antibodies failed to detect an interaction with EscU or non-cleaving EscU variants (Figure 6). Although we cannot rule out the possibility of a direct CesT-EscU interaction, we provide evidence that efficient CesT membrane association occurs when EscU is auto-cleaved (Figure 5A).

It has been demonstrated that the YscU/FlhB proteins interacts with multiple components within their respective T3SS [24,60-62]. A shortlist of protein interactions includes YscI, YscK, YscL, YscN, YscQ and YscV (using the *Yersinia *nomenclature) among other proteins. The putative YscL, YscI and YscQ homologues within the EPEC LEE PAI are believed to be Orf5, rOrf8 and SepQ respectively [63] although the homology scores are very low (below 15%). A yeast two hybrid screen identified rOrf8 (putative YscI homologue) as an EscU binding partner [64]. The YscI/PrgJ family form an inner rod within the T3SS needle complex, a structure that may exist for EPEC but has not been identified in highly purified needle preparations [20]. In *Shigella*, Spa32 is known to interact with the C-terminal region of Spa40 [40], however in EPEC an evident Spa32 homologue has not been identified. The notable absence of clear homologues for known YscU protein partners is puzzling although might be explained by the different architecture of the EPEC T3SS compared to *Salmonella*, *Shigella *and *Yersinia*. Specifically, a long polymeric filament composed of EspA sits atop the EPEC needle complex [21].

From the various crystal structures now available, it has been hypothesized that the conserved auto-cleavage mechanism for the YscU/FlhB group of proteins results in a critical surface to promote protein interactions for secreted substrates [26-29]. We extend this interpretation with experimental data to further suggest that EscU auto-cleavage promotes translocon and effector protein secretion presumably by acting at the base of the EPEC T3SS.

## Conclusions

This study provides evidence that intermediate phenotypes can be identified in the EPEC T3SS secretory pathway suggesting that sequential binding events are involved in type III effector translocation into host cells. The conserved mechanism of auto-cleavage, shown here for EscU, is a critical event that supports type III effector translocation. Additional studies will be required to identify the temporal sequence of events and to functionally characterize how protein substrates are trafficked through the T3SS.

## Methods

### Bacterial Strains and Growth Media

Bacterial strains generated and used in this study are listed in Table 1. Bacterial strains were routinely cultured in Luria broth (LB) (1% [w/v] tryptone, 0.5% [w/v] yeast extract, 1% [w/v] NaCl) at 37°C. Antibiotics (Sigma) were added when appropriate, to a final concentration of 50 μg/ml kanamycin, 50 μg/ml streptomycin, 30 μg/ml chloramphenicol, 200 μg/ml ampicillin, 10 μg/ml tetracycline.

**Table 1:** Strains and plasmids used in the study

Strains	Description	Source/comment
Wild type EPEC	EPEC strain E2348/69, streptomycin-resistant, BFP positive.	[35]
*ΔescU*	*escU *deletion mutant	This study
*ΔsepD*	*sepD *deletion mutant	
*ΔsepDΔescU*	Double mutant derived from *ΔsepD*	This study
*ΔsepD::escU(N262A)*	Cis-complemented *ΔsepDΔescU *strain	This study
*ΔsepD::escU(P263A)*	Cis-complemented *ΔsepDΔescU *strain	This study
*ΔsepDΔtir*	Double mutant derived from *ΔsepD*	[35]
*ΔescNΔescU*	Double mutant derived from Δ*escN*	This study
SM10λ*pir*	*E. coli *strain that is permissive for pRE112 replication	
DH5α	*E. coli *strain used for cloning	
DH5αλ*pir*	*E. coli *strain used for cloning, permissive for pRE112 replication	
**Plasmids**		
pET21 a+ pACYC184	T7/HIS tagged fusion vector Broad host range plasmid, P15A derived	Novagen [72]
pETescU_HIS_	pET21a+ expressing EscU-HIS	This study
pETescU(N262A)_HIS_	pET21a+ expressing EscU(N262A)-HIS	This study
pETescU(P263A)_HIS_	pET21a+ expressing EscU(P263A)-HIS	This study
pEscN	pACYC184 expressing EscN	[65]
pFLAG-CTC	Cloning vector to express C-terminal FLAG fusion proteins from tac promoter	Sigma
pJLT21	pACYC184 expressing EscU-HIS	This study
pJLT22	pACYC184 expressing EscU(N262A)-HIS	This study
pJLT23	pACYC184 expressing EscU(P263A)-HIS	This study
pJLT24	pFLAG-CTC backbone vector that expresses a HA-EscU-FLAG tagged protein	This study
pJLT25	pFLAG-CTC backbone vector that expresses a HA-EscU(N262A)-FLAG tagged protein	This study
pJLT26	pFLAG-CTC backbone vector that expresses a HA-EscU(P263A)-FLAG tagged protein	This study
pRE112	λ*pir *origin, suicide vector encoding *sacB*	[73]
PΔescU	*escU *deletion fragment cloned into pRE112 suicide vector	This study
pTir-bla	pCX341 expressing a Tir-TEM1 fusion protein	This study

### Isolation of Genomic and Plasmid DNA

Genomic DNA was isolated from bacterial strains using the Purogene genomic DNA isolation kit (Gentra systems). Plasmids were isolated from bacterial strains using the QIAprep spin miniprep kit (Qiagen).

### Recombinant Plasmid Construction

The *escU *gene was amplified via polymerase chain reaction (PCR) from EPEC genomic DNA using primers JT8 and JT10 and cloned into pET21a+ to create pET*escU*_HIS _(see table 2 for the sequences and list of primers used in this study). This construct overexpresses EscU-His from the recombinant T7 promoter. The pET*escU*(N262A)_HIS _and pET*escU*(P263A)_HIS _vectors were generated using the Phusion site directed mutagenesis kit (Finnzymes), following the manufacturer's directions. Briefly, primers pairs JT12/JT13 and JT14/JT15, that had phosphorylated 5' ends, were used to generate amplicons of pET*escU*(N262A)_HIS _and pET*escU*(P263A)_HIS _using pET*escU*_HIS _as template. The sequences of pET*escU*_HIS_, pET*escU*(N262A)_HIS _and pET*escU*(P263A)_HIS _were verified using the universal T7 forward and reverse primers that flank the pET21a+ multiple cloning site by sequencing.

**Table 2:** Primers used in this study

Primer	Sequence (5'-3')
HAEscU	CCGCTCGAGTACCCATACGATGTTCCAGATTACGCTATGAGTGAAAAAACAGAAAAGCCC
EscURevBglII	GAAGATCTATAATCAAGGTCTATCGCAATACG
JT1	CCGAGCTCGTTACAGGATCAAACATTGCC
JT2	GCGCTAGCTTCACTCATTAATCATGCTCGG
JT3	CCGCTAGCCTTGATTATTAATCGATAATTTGC
JT4	GCCTCGTGGGCAATATCATTGCG
JT7	CCAAATGCAGTAGAACTCAGAAGGC
JT8	GGGGATCCCTGACATAATTGATAGATCGTTACCG
JT10	ACATGCATGCTCAGTGGTGGTGGTGGTGGT
JT12	/PHOS/GTTATTGTTAAAGCCCCGACTCACATT
JT13	/PHOS/GTTTGATTTTTTGATGTTATTCGC
JT14	/PHOS/GTTAAAAACGCGACTCACATTGCG
JT15	/PHOS/AATAACGGTTGATTTTTTGATGTTATT
NT278 NT279 NT281 NT282	AAGGCGCCTTTTTAACAATAACGGTTGA AAGGCGCCGACTCACATTGCGATTTGCCTA GCGACTCACATTGCGATTTGCCTA GTTTTTAACAATAACGGTTGATT
XH1	CCATTAATATGTCTACAGGAGCATTAGG
XH2	CGGAATTCTCAACGAAACGTACTGGTCC

/PHOS/indicated primers that are 5' phosphorylated, restriction sites have been underlined.

To generate pJLT21, pJLT22 and pJLT23, DNA fragments corresponding to the relevant *escU *allele were amplified by PCR using primers JT8 and JT10 from isolated plasmid DNA of pET*escU*_HIS_, pET*escU*(N262A)_HIS _and pET*escU*(P262A)_HIS_, respectively. The resulting 1.5 kb products were purified and treated with restriction enzymes and cloned into pACYC184 treated *Bam*HI and *Sal*I. To generate pJLT24, pJLT25 and pJLT26, DNA fragments corresponding to the relevant *escU *allele were amplified by PCR using primers HAEscU and EscURevBglII using plasmid DNA as template. The resulting PCR products were purified and sub-cloned into pFLAG-CTC vector using *Xho*I and *Bgl*II.

To generate pTir-bla, primers XH1 and XH2 were used to PCR amplify the *tir *open reading frame (without the stop codon) using EPEC genomic DNA as template. The resulting PCR product was treated with *Ase*I and *Eco*RI and cloned into *Nde*I/*Eco*RI treated pCX341 (generously provided by I. Rosenshine) [43] to create pTir-bla. The resulting plasmid construct was electroporated into EPEC and transformants were selected using tetracycline. Expression of Tir-TEM1 was verified by immunoblotting using anti-TEM1 antibodies (QED Biosciences).

### Construction of mutants in EPEC E2348/69

A chromosomal deletion of *escU *was generated using allelic exchange [39]. Chromosomal DNA regions flanking the *escU *open reading frame were amplified from EPEC genomic DNA by PCR using primer pairs JT1/JT2 and JT3/JT4. The resulting 0.9 kb and 1.2 kb products were treated with *Nhe*I and then combined in a 1:1 ratio followed by the addition of T4 DNA ligase. After an overnight incubation at 16°C, an aliquot of the ligation reaction was then added to a PCR with primers JT1 and JT4 which generated a 2.1 kb product. The product was digested with *Sac*I and cloned into pRE112 using *E. coli *DH5αλ*pir *as a cloning host. The resulting plasmid PΔ*escU *was verified using primers JT1 and JT4 by sequencing. PΔ*escU *was then transformed into the conjugative strain SM10λ*pir *which was then mated with EPEC E2348/69. EPEC integrants harbouring PΔ*escU *on the chromosome were selected by plating onto solid media supplemented with streptomycin and chloramphenicol. The resulting colonies were then plated onto sucrose media (1% [w/v] tryptone, 0.5% [w/v] yeast extract, 5% [w/v] sucrose and 1.5% [w/v] agar) and incubated overnight at 30°C. The resulting colonies were screened for sensitivity to chloramphenicol, followed by a PCR using primers JT1 and JT7 to verify deletion of the *escU *from the chromosome. Cis-complementation mutants were generated using the same allelic exchange approach using primers NT278 and NT279 for *escU*(N262A) and primers NT281 and NT282 for *escU*(P263A) genetic constructs. To generate the *ΔescNΔescU *and *ΔsepDΔescU *double mutants, SM10λpir/PΔ*escU *was conjugated with *ΔescN *[65], *ΔsepD *[66] as described above. For genetic trans-complementation studies, the appropriate plasmids were transformed into electrocompetent strains followed by antibiotic selection.

### *In vitro *secretion assay

Secretion assays were performed as previously described [39] with some minor modifications. To aid in the precipitation of proteins from secreted protein fractions, bovine serum albumin (100 ng) was added as a carrier protein during the precipitation step.

### Isolation of bacterial membrane fractions

Total crude bacterial membrane preparations were generated from EPEC strains using a previously established protocol that preserves peripherally associated proteins and enriches for T3SS needle complexes [39]. Fractionation of membrane preparations was achieved using sucrose density gradients as previously described [39].

### Immunoprecipitation

Immunoprecipitations with EPEC cell lysates were performed as previously described [39]. Briefly, 500 ng of affinity purified polyclonal anti-CesT antibody was added to 50 μl of Protein A conjugated agarose beads (Invitrogen) followed by washing as directed by the manufacturer. The antibody-bead mixture was blocked in phosphate buffered saline (PBS, 137 mM NaCl, 2.7 mM KCl, 4.3 mM Na_2_HPO_4_, 1.47 mM KH_2_PO_4_) supplemented with 1% (w/v) bovine serum albumin and then added to lysate preparations and incubated overnight at 4°C on a rotator. The samples were gently pelleted and the agarose beads were washed 3 times with PBS. The agarose beads were then exposed to 100 mM glycine (pH 2.2) to elute bound proteins and neutralized with 1 M Tris (pH 8.8) and then prepared for SDS-PAGE.

### Infection of HeLa cells

HeLa cells [American Type Culture Collection (ATCC)] were seeded onto sterile glass coverslips at a density of 1 × 10^5 ^/ml, grown for 24 hrs and then infected with various EPEC strains at a multiplicity of infection of 50 for 3 hours. The infected HeLa cells were then prepared for microscopy as previously described [35]. Images were detected using a Zeiss Axiovert 200 inverted microscope and captured using a Hamamatsu ORCA-R2 digital camera. Microscopy based quantification of EPEC intimate adherence (binding index) was performed as previously described [67]. Briefly, GFP positive bacteria (which were identified by GFP fluorescence) that were associated with actin pedestals were quantified. At least 50 cells were examined per sample.

### β-lactamase reporter assays

Type III effector-TEM1 fusion reporter assays for EPEC strains were performed as previously described [42] with minor modifications. Briefly, HeLa cells (seeded to confluence in 96 well, black, clear bottom plates [Costar 3603]) were infected with a MOI of approximately 50 for 2 hours using bacteria that had been pre-activated in DMEM +10% FBS for 2 hours at 37°C, 5% CO_2_. After 1 hour of infection, IPTG was added to a final concentration of 0.5 mM. The infected cells were gently washed twice with DMEM and then loaded with CCF2/AM using a Toxblazer kit (Invitrogen). The 96 well plate was incubated for 90 min in the dark and then placed in a Victor X plate reader (Perkin Elmer) set to read fluorescence using an excitation filter for 405 nm and emission filters for 460 nm (blue signal)/530 nm (green signal). Blue/green signal ratios and statistical significance (two sided Student's t test) were calculated as previously described [42]. The presented data are mean values of the results from three experiments.

### Protein electrophoresis and Immunoblotting

All protein samples were separated by SDS-PAGE as described [68]. Separated polypeptides were visualized by Coomassie G-250 blue staining or Sypro Ruby Red (Bio-Rad, according to manufacturers directions). Pre-stained Broad Range Protein Markers (New England Biolabs, cat. # 7708) were used for standards. For immunoblotting, separated polypeptides were transferred to Immobilon-P membrane (Millipore), blocked with skim milk (5% [w/v] in Tris buffered saline + Tween20 [TBS-T]) or BSA, and then incubated with specific antibodies at working concentrations; anti-EscJ, 1:500 [69]; anti-EscN, 1/500 [39]; anti-EspB, 1:200 [70]; anti-EspA [71]; anti-intimin 1:1000 (gift from J. Leong); anti-HA, 1:5000 (gift from R. Duncan); anti-FLAG, 1:5000 (Sigma); anti-DnaK, 1:5000 (Calbiochem), anti-Tir, 1:1000 [35]; anti-TEM1, 1:2000 (QED Biosciences); goat anti-mouse conjugated to horse radish peroxidise (HRP), 1:5000 (Rockland immunochemicals); goat anti-rabbit conjugated to HRP, 1:5000 (Rockland immunochemicals); goat anti-rat conjugated to HRP, 1:5000. Anti-CesT polyclonal antibodies were raised in New Zealand white rabbits against a synthetic peptide (LENEHMKIEEISSSDNK) corresponding to the C-terminal region of CesT (Pacific Immunology, CA, USA, [NIH Animal Welfare Assurance Number: A4182-01]). Final bleeds were affinity purified against the peptide by the supplier and used in immunoblots at a 1:10000 dilution. Immunoblots were developed using an enhanced chemiluminescence reagent (ECL, GE Healthcare) and data captured on a VersaDoc 5000 MP (Bio-Rad). Densitometry measurements to evaluate band intensity from chemiluminescent signals in immunoblotting experiments were performed using Quantity One software (Bio-Rad). Immunoblots were imaged simultaneously and within exposure times that were within an empirically determined linear range of signal detection.

### Ethics statement

The grant proposal supporting this research was reviewed by the Dalhousie University Ethics Officer. Ethics approval was not required as the research does not involve human subjects, primary human cell lines/samples or animals.

## Authors' contributions

JLT performed cloning, secretion and infection assay experiments. XH constructed pTir-TEM1 fusions. NAT performed secretion, infection, effector translocation and sub-cellular fractionation assays. JLT and NAT designed experiments and wrote the paper. JLT, XH and NAT have read and approved the final version of the manuscript.
